# User Experience With a Personalized mHealth Service for Physical Activity Promotion in University Students: Mixed Methods Study

**DOI:** 10.2196/64384

**Published:** 2025-03-28

**Authors:** Silke Wittmar, Tom Frankenstein, Vincent Timm, Peter Frei, Nicolas Kurpiers, Stefan Wölwer, Axel Georg Meender Schäfer

**Affiliations:** 1 Faculty of Social Work and Health HAWK University of Applied Sciences and Arts Hildesheim/Holzminden/Göttingen Hildesheim Germany; 2 Faculty of Design HAWK University of Applied Sciences and Arts Hildesheim/Holzminden/Göttingen Hildesheim Germany; 3 Institute for Sport Science University of Hildesheim Hildesheim Germany

**Keywords:** usability testing, health promotion, exercise, smartphone app, mHealth, physical activity, user experience, user, university student, undergraduate, college, student, mixed methods, physical fitness, digital intervention, mobile health, promote, engagement, mobile phone

## Abstract

**Background:**

Regular physical activity (PA) is known to offer substantial health benefits, including improved physical fitness, reduced risk of disease, enhanced psychological well-being, and better cognitive performance. Despite these benefits, many university students fail to meet recommended PA levels, risking long-term health consequences.

**Objective:**

This study evaluated the user experience (UX) of futur.move, a digital intervention aimed at promoting PA among university students. The service delivers personalized, evidence-based content to foster sustained engagement in PA.

**Methods:**

A mixed methods approach was used to evaluate the prototype of futur.move. UX assessments included on-site and online user tests, standardized questionnaires, and online focus groups. A total of 142 university students participated, with 23 joining additional focus groups. Each participant tested the service for 30 minutes. Quantitative data were collected using the User Experience Questionnaire and analyzed descriptively, followed by correlation analysis with variables such as PA level, age, gender, and experience with PA apps. Qualitative insights were gathered from transcribed focus group discussions and analyzed using content-structuring, qualitative content analysis. Quantitative findings were cross-validated with qualitative data.

**Results:**

The UX received positive ratings across 4 User Experience Questionnaire scales (range –3 to +3; higher numbers indicate positive UX): attractiveness (median 1.67, IQR 1.04-2.17), perspicuity (median 1.5, IQR 0.5-2), stimulation (median 1.5, IQR 1-2), and novelty (median 1.25, IQR 0.5-2). Weak correlations were found between adherence to World Health Organization guidelines for PA and the perspicuity subscale (η=0.232, *P*=.04), and between age and the perspicuity (Kendall τb=0.132, *P*=.03) and stimulation subscales (Kendall τb=0.144, *P*=.02), and a moderate correlation was found between gender and the novelty subscale (η=0.363, *P*=.004). Critical feedback from focus group discussions highlighted issues with manual data entry. Qualitative findings aligned with the quantitative results, emphasizing students’ appreciation for the personalized, diverse content and social networking features of futur.move.

**Conclusions:**

futur.move demonstrates favorable UX and aligns with student needs, particularly through its personalized content and social features. Improvements should focus on reducing manual data entry and enhancing feature clarity, particularly for the features “your condition” and “goal setting.” While correlations between UX ratings and demographic variables were weak to moderate, they warrant further investigation to better address the diverse target audience. The feedback from the students serves as a basis for further adapting the service to their needs and expectations. Future work will involve coding an advanced prototype and conducting a longitudinal study to assess its impact on PA behavior and sustained engagement.

## Introduction

The words commonly attributed to Hippocrates, “walking is humanity’s best medicine,” are more relevant than ever. In an era dominated by sedentary lifestyles, university students could greatly benefit from incorporating more physical activity (PA) into their daily routines.

Regular PA offers many important health benefits, including improved muscular and cardiorespiratory fitness, reduced risk of diseases, and enhanced psychological well-being [1-3]. PA also positively affects sleep and cognitive performance, including academic achievements and attention [1,3-5]. PA can also improve social skills and self-confidence [6].

The World Health Organization (WHO) recommends at least 150 minutes of moderate- to vigorous-intensity aerobic PA or at least 75 minutes of vigorous-intensity aerobic PA and muscle-strengthening activities of moderate to vigorous intensity for all major muscle groups on 2 or more days a week for adults [3]. Despite the benefits of regular PA, only a minority of young adults, including university students, meet the recommended PA levels, with 81% of adolescents [7] and 28% of adults worldwide and 42% in high-income Western countries [8] failing to adhere to these guidelines. Furthermore, studies show that students’ PA levels drop significantly during examination periods and that they are more active during weekdays than on weekends [9-11]. As students undergo significant life transitions, their lifestyle habits, including PA patterns, can persist into adulthood if not addressed [12,13]. Therefore, universities face the challenge of supporting students in adopting more active lifestyles [14].

Promoting PA through mobile health (mHealth) services holds great potential, especially as students are frequent users of technology [15]. mHealth interventions are preferred by young people over traditional face-to-face approaches [16]. However, many current apps often fail to incorporate evidence-based recommendations for PA promotion, with developers prioritizing marketing strategies over addressing real user needs [17,18].

Positive user experience (UX) influences user satisfaction, promoting sustained product use and increasing recommendations [19]. “To create successful products or services it is necessary to ensure that the product has a sufficiently high user experience” [20]. UX is defined as “...a person’s perceptions and responses that result from the use or anticipated use of a product, system or service. Users’ perceptions and responses include the users’ emotions, beliefs, preferences, perceptions, comfort, behaviors, and accomplishments that occur before, during and after use” [21]. Evaluating UX early in the prototyping phase allows developers to incorporate user feedback and refine features to meet diverse user needs [20,21]. In the context of PA services, successful interventions include behavior change techniques, such as self-monitoring, goal setting, behavioral feedback, social support, and social comparison, to effectively increase PA [22-26]. The challenge remains to maintain long-term user engagement with mHealth interventions [16]. Numerous studies have identified poor functionality and technical issues as barriers to engagement with mobile interventions, while design features such as personalization, social interaction, and customized feedback enhance user engagement [16]. Personalization is crucial, as students’ PA needs vary widely [6], and one-size-fits-all approaches are inadequate [27-29].

This study aims to comprehensively evaluate the UX of the futur.move prototype, an mHealth service designed to enhance PA among university students. Specifically, this study will focus on the following UX aspects: (1) attractiveness—whether users like the service, (2) perspicuity—whether the service is easy to learn, (3) stimulation—whether using the service is exciting and motivating, and (4) novelty—whether the service is innovative and creative. The central research question is: “How do students evaluate the UX of the futur.move prototype?” Of particular interest are the specific improvements students suggest to optimize implementation.

## Methods

### Study Design

This study design follows a sequential approach with a series of cross-sectional surveys using structured online questionnaires with qualitative focus group discussions to comprehensively evaluate the futur.move prototype. On-site tests were conducted with a large participant group at the university, while smaller online sessions were held via Zoom (Zoom Video Communications Inc).

This study report follows the CHERRIES (Checklist for Reporting Results of Internet e-Surveys) reporting guideline for reporting web-based surveys [30] (Multimedia Appendix 1).

### futur.move

futur.move is a digital service that aims to improve students’ PA through a smartphone app and website in the form of a progressive web application. According to a definition of service design, a service should be useful, usable, and desirable for the customer, while being effective, efficient, and unique for the provider. Services include interactions and interfaces between the customer and the provider [31].

**Figure 1:**
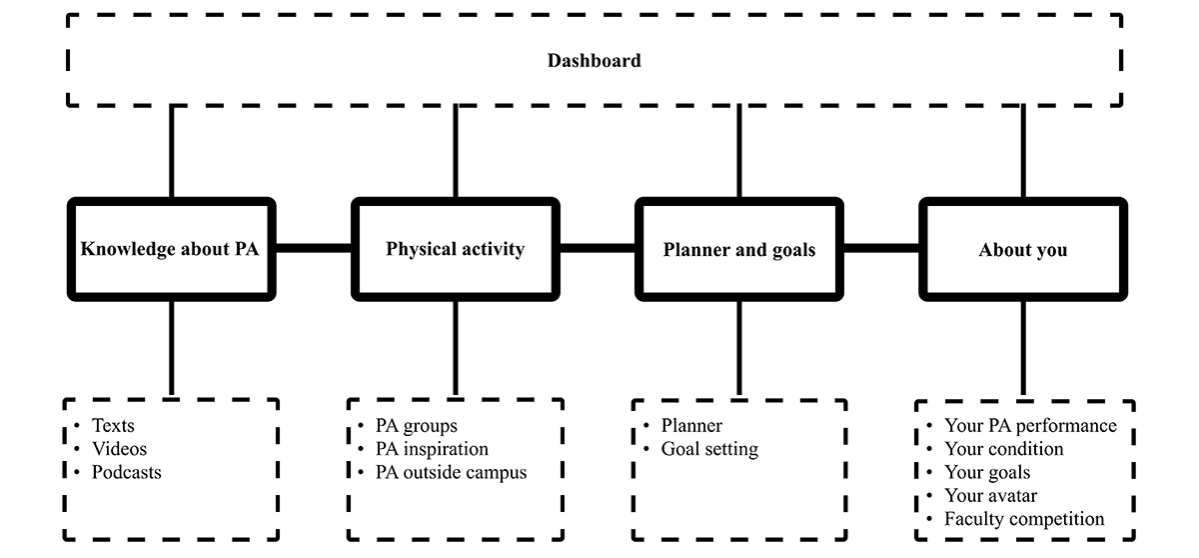
Structural overview of the features and content offered by the futur.move mHealth service. PA: physical activity. mHealth: mobile health.

The development of futur.move follows the holistic framework for the development of eHealth technologies [32] and includes 5 phases: context analysis, design, UX evaluation, programming, and long-term implementation. Currently, the prototype is in the UX evaluation phase (phase 3). The design is informed by a literature review, 2 student creative workshops (n=13 participants), and social cognitive theory [33]. Studies report that PA interventions are more successful when they are theory based [34,35]. Components of social cognitive theory such as health-related knowledge, individual goal setting, and social support are transferred into the functions of the app to support users in successfully and sustainably increasing their PA. An interdisciplinary team of UX designers and health scientists developed the app iteratively in the collaborative design tool Figma (Figma Inc, 2022) with active involvement from the target audience. Figure 1 shows the content structure of futur.move features.

**Figure 2:**
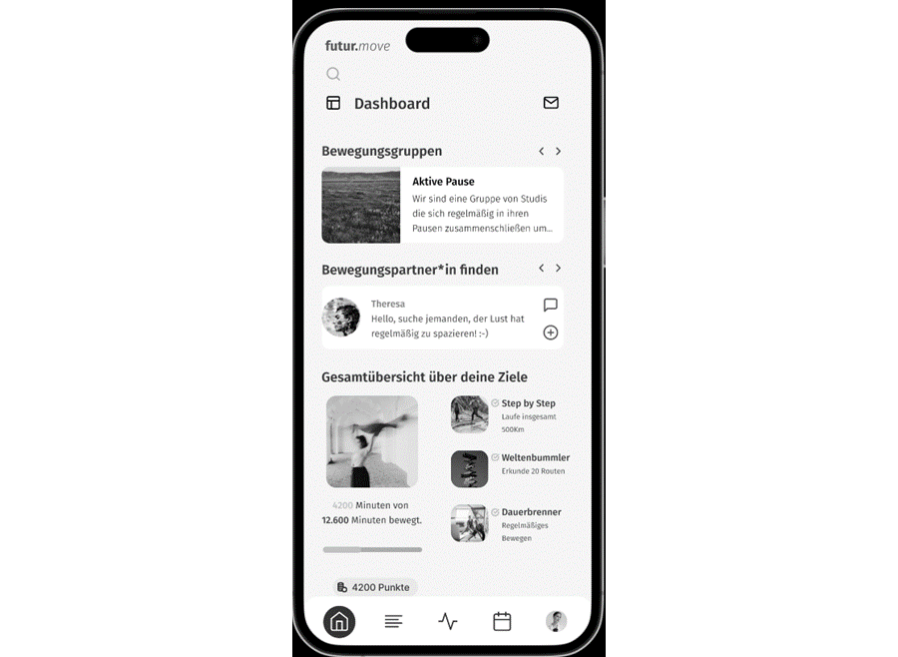
Screenshot of the customizable dashboard of the futur.move mHealth service as viewed on a smartphone. mHealth: mobile health. An English version of the dashboard is available in Multimedia Appendix 2.

After registration and reporting PA habits via the European Health Interview Survey–Physical Activity Questionnaire (EHIS-PAQ) [36], users access personalized content across five areas: (1) “dashboard,” (2) “knowledge,” (3) “physical activity,” (4) “planner and goals,” and (5) “about you” (Figure 1). A messaging function is also available. In the “knowledge” section, users access PA-related information through various formats such as text, videos, and podcasts. The “physical activity” section enables users to find inspiration for PA, join or create PA groups, and use links to find PAs outside the campus. “Planner and goals” offers a calendar view and goal-setting features, while “about you” manually tracks PA habits and, goals, and provides a faculty competition. By participating in PA, users can earn points for their faculties and win rewards such as a faculty party. Figure 2 shows the personalizable dashboard of futur.move.

### Participants and Recruitment

User testing was conducted from October 27 to December 21, 2023, at University of Applied Sciences and Arts Hildesheim/Holzminden/Göttingen (HAWK; N=6300 students in winter 2022/2023).

The convenience sample for this study was recruited from the entire student body of HAWK. For the on-site sessions, a high number of participants was targeted to enable valid statistical analyses. Therefore, researchers reached out directly to professors from all 3 university campuses and various faculties, requesting their support in implementing this study within their seminars. On-site sessions were finally held in 4 seminars across different faculties and locations. Participation in this study was integrated into the respective seminars; however, it remained entirely voluntary for all participants, ensuring that their consent was informed and given freely without any obligation.

For the online sessions with focus group discussions, all students at HAWK were invited via email, flyers, and the university’s social media channels. Flyers were posted in high-traffic areas on the university campuses, such as the cafeteria and the library. Additionally, first-year students from the Faculty of Social Work and Health were personally approached at a welcome event for new students. Due to low participation, students were also approached at a campus café. The latter method of reaching out to students proved to be the most effective. A link distributed through the mentioned media allowed registration via HAWK’s online learning tool, followed by contact with the project team, provision of information, and signing of the consent form. Only those who returned the signed form participated. To participate in both the on-site and online data collection, inclusion criteria were enrollment and no prior involvement in futur.move.

Recommended sample sizes for quantitative UX testing to estimate key performance parameters range from 10 to 1689 participants, depending on the margin of error. “A sample size of 115 at 90% confidence can detect a 10% significant difference” [37]. Therefore, we aimed to recruit a minimum of 65 participants for the survey to achieve 90% confidence. For qualitative UX testing, it is recommended to conduct focus groups with 4 to 37 participants to identify usability issues. A sample size of 18 is needed to detect a 10% insight occurrence [37]. Consequently, we aimed to recruit at least 18 participants for the focus group discussions. Given the maximum group size of 6 to 8 participants per focus group [38], we planned for a minimum of 3 groups.

### Ethical Considerations

Ethical approval was obtained from the HAWK ethics committee on September 29, 2023. All participants gave written informed consent before participation, agreeing to the use of deidentified data for secondary analyses and the publication of results. Data were collected anonymously, and no personally identifiable information was included in the final dataset. Participation was voluntary. Participants could withdraw at any time without justification. Data were stored on the university’s server for secure keeping until January 10, 2033, and will then be deleted by the researchers. Throughout this study, only deidentified data were used, and access was restricted solely to the research team. Participants in the online sessions received a €10 (US $10.85) voucher for an online retailer.

### Procedures

#### On-Site Data Collection

Quantitative data were collected on-site during the seminars with a project team member present. Participants assumed the role of “Kim,” a fictitious user of futur.move, to understand prestored data and experience all app features. After an introduction and onboarding, they received a task sheet with 6 tasks to independently test the service for approximately 30 minutes (eg, finding exercise information, Multimedia Appendix 3). They accessed the prototype via a device (eg, laptop or tablet).

After completing each task, participants provided initial feedback on the task sheet and then completed the online questionnaire. This included sociodemographic data, PA behavior (EHIS-PAQ) [36], and usability ratings using the User Experience Questionnaire (UEQ) [39]. The survey, hosted via SoSci Survey (version 3.5.02), presented the items to each participant in the same order, allowed participants to change their answers using a back button, and omitted irrelevant questions. There was no check for completeness before submission. No cookies were used. The online survey consisted of 22 pages, including the introduction and conclusion. The number of items on each page varied. Each participant used a unique ID to merge task sheet responses with the online survey for analysis. The ID was randomly assigned on each task sheet. Each code could only be used once. On-site sessions lasted approximately 60 minutes per session.

A pretest was conducted to ensure clarity and feasibility. Four students tested the task sheets, while 2 project members and 2 individuals from the target group tested the online survey. Feedback led to revisions of both instruments.

#### Online Data Collection

Online data collection was conducted via the Zoom videoconferencing tool with 2 project team moderators. Participants registered for user tests, and sessions were scheduled based on availability. Participation was voluntary, and participants could withdraw at any time.

The first part of the online session mirrored the on-site data collection. The moderators reviewed task sheets and survey feedback to refine focus group discussions, gathering targeted qualitative insights beyond the prepared questions.

### Measures

#### Demographics

As part of the online survey, participants provided their age, gender, height, weight, faculty affiliation, and whether they had used apps to improve PA before. Additionally, the EHIS-PAQ [36] was used to collect standardized data on students’ PA. The EHIS-PAQ is a domain-specific instrument for measuring (1) work-related PA, (2) transport-related PA, and (3) leisure-time PA. It distinguishes between “aerobic” and “muscle-strengthening” PA and allows the assessment of compliance with health-promoting PA recommendations [36].

#### About UX

UX was assessed using the UEQ [39]. The UEQ is a quantitative instrument for assessing the UX of interactive products. It originally consisted of 6 subscales with a total of 26 items with classic usability aspects (ie, efficiency, perspicuity, or dependability) and UX aspects (ie, originality or stimulation). The authors state that entire subscales can be omitted if they are irrelevant to the investigation [40]. Consequently, the “efficiency” and “dependability” scales from the original questionnaire were not included in the analysis, as no valid results could be expected for these scales at the stage of development of the service. In this study the items of the four subscales (1) attractiveness (do the users like the service or not?), (2) perspicuity (is it easy to learn how to use the service?), (3) stimulation (is the use of the service exciting and motivating?), and (4) novelty (is the service innovative and creative?) were used. The attractiveness scale is represented by 6 items, while the other scales are represented by 4 items each. The items are semantic differentials, for example, attractive and unattractive. They can be rated on a 7-point scale from –3 (horribly bad) to +3 (extremely good). Scale scores are average item ratings across participants. Scores between –0.8 and 0.8 represent a neutral evaluation of the scale, scores>0.8 represent a positive evaluation, and scores<0.8 represent a negative evaluation [40].

#### Evaluation of Individual Features

On the task sheet for testing futur.move, students had the opportunity to indicate how much they liked each feature of futur.move. They could tick a value from very good (1) to poor (5) on a 5-point Likert scale. Within this framework, the students rated the five features: (1) faculty competition, (2) your condition, (3) knowledge, (4) PA group (hiking), (5) PA inspirations, and (6) goal setting. Additionally, the online questionnaire asked students how they would rate futur.move overall on a 5-point Likert scale from 1 (very good) to 5 (poor).

#### Focus Groups

The focus groups were conducted to qualitatively summarize the quantitative feedback from the students. The questions in the focus groups were, therefore, related to the quantitative part of the respective user tests. Follow-up questions were individually prepared by the moderators by reviewing the anonymized data from the user tests. The focus group structure followed the recommended procedure in empirical social science [41]. The duration of the focus groups was approximately 120 minutes, and they were conducted by VT and SW, who moderated the discussion. The discussion was recorded using the recording feature of Zoom.

The first question in the focus groups was what participants remembered most about futur.move. Following this, an overview of the UEQ scales was briefly presented. Based on the UEQ scores, questions about divergences in the scoring of individual scales were discussed. Students were asked to describe their quantitative assessments in more detail, and discrepancies in the service ratings from the task sheet were examined. Finally, there was an opportunity for participants to report any further requirements. Discussions were recorded with participants’ consent for later transcription and stored on the university’s server.

### Data Analyses

#### Quantitative Data Analyses

The quantitative data from the questionnaires were analyzed using IBM SPSS (version 27; IBM Corp).

The UEQ Data Analysis Tool (version 12) transformed the UEQ data, which were then merged in SPSS using participant IDs. Meta-information was added, and variable labeling was adjusted, including scale levels [42]. Data cleansing involved checking for incorrect or implausible information and marking missing values with the code 999. Questionnaires with missing values were included in the analysis.

For the UEQ data, items on the subscales were analyzed for divergent ratings. Cases were recommended for exclusion if at least 3 subscales were conspicuous or if more than 15 of 26 items had identical ratings. No records were excluded based on plausibility, but 8 records from the task sheets were excluded due to missing online survey data.

As the UEQ data were not normally distributed, the median and IQR were calculated. Nonetheless, scale means, variances, and Cronbach α were calculated using the UEQ Data Analysis Tool, to use the provided benchmark for categorizing scale scores against data from 468 studies on various products, rating them from “poor” to “excellent” [40].

In addition to the descriptive data analysis, a correlation analysis was conducted to explore the relationships between the UX evaluation of the futur.move, as measured by the UEQ, and various demographic and usage-related factors. These factors included compliance with WHO guidelines for PA, age, gender, and prior experience with apps aimed at promoting PA.

Associations were analyzed using the η and Pearson chi-square tests for metric and nominal-scaled data. For associations between 2 metric variables, Kendall τb was used. The significance level for all tests was set at α≤.05.

#### Qualitative Data Analyses

The focus group recordings were automatically transcribed verbatim using the tool noScribe (version 0.4.1). The transcripts were then manually corrected and anonymized, with participant names replaced by the IDs matching those from the quantitative survey, allowing data linkage.

Two project members analyzed the transcripts using MAXQDA 2022 (VERBI-Software. Consult. Sozialforschung GmbH 2021) following content-structuring, qualitative content analysis [43]. The data were analyzed based on the question: “How do students evaluate the futur.move prototype?” with the 4 UEQ scales (attractiveness, perspicuity, stimulation, and novelty) used as deductive categories. Further inductive categories could be formed based on the material, but no additional categories emerged. Subcategories of the main categories were formed inductively during the analysis.

## Results

### Quantitative Findings

#### Demographics

A total of 142 people (mean age 24, SD 4 years; female: n=105, 74%; male: n=33, 23%) participated in the user tests. Change to: A total of 142 people (mean age 24, SD 4 years; female: n=105, 75%; male: n=33, 23%) participated in the user tests. Of these, 23 also took part in the focus groups. Most participants were students from the Faculty of Social Work and Health (n=60, 42%). Approximately a third of the students had prior experience using an app to promote PA (n=49, 35%). The majority of students (n=87, 61%) met the WHO guideline of practicing more than 150 minutes of moderate aerobic activity per week, while 49% (n=69) met the guideline of doing muscle-strengthening exercises at least 2 days a week. Overall, 40% (n=57) of participants met the WHO guidelines for PA, fulfilling both criteria. The mean BMI of the students was 23 (SD 4), which falls at the upper end of the normal weight range. Table 1 summarizes the information provided by the students.

**Table 1:** Demographics of university students participating in user tests conducted from October 27 to December 21, 2024.

Characteristics		Participants
Age (years), mean (SD)		24 (4)
**Gender, n (%)**		
	Female	105 (74)
	Male	33 (23)
	Diverse	1 (1)
	Not specified	3 (2)
**Faculty, n (%)**		
	Social work and health	60 (42)
	Design	26 (18)
	Engineering and health	22 (16)
	Architecture, engineering, and conservation	20 (14)
	Management, social work, and construction	13 (9)
	Not specified	1 (1)
“**Do you already use digital services to promote physical activity?” n (%)**		
	Yes	49 (35)
	No	74 (52)
	Not specified	19 (13)
BMI (kg/m^2^), mean (SD)		23 (4)
**Fulfillment WHO^a^ guideline >150 minutes of moderate aerobic activity, n (%)**		
	Yes	87 (61)
	No	55 (39)
**Fulfillment WHO guideline ≥2 days/week muscle strengthening, n (%)**		
	Yes	69 (49)
	No	73 (51)
**Fulfillment WHO guideline for physical activity overall, n (%)**		
	Yes	57 (40)
	No	85 (60)

^a^WHO: World Health Organization.

#### futur.move UX

The UX of futur.move is indicated by the average score on each respective scale. As the data are not normally distributed, the median, IQR, and the minimum and maximum range are also reported. Attractiveness received a median score of 1.67 (IQR 1.04-2.17) and a mean score of 1.55 (SD 0.91) on a scale from –3 to +3. Perspicuity was rated with a median of 1.5 (IQR 0.5-2) and a mean of 1.28 (SD 0.97), stimulation with a median of 1.5 (IQR 1-2) and a mean of 1.37 (SD 1.03), and novelty with a median of 1.25 (IQR 0.5-2) and a mean of 1.27 (SD 0.98). Positive ratings are indicated by scores greater than 0.8 (UEQ manual).

**Figure 3:**
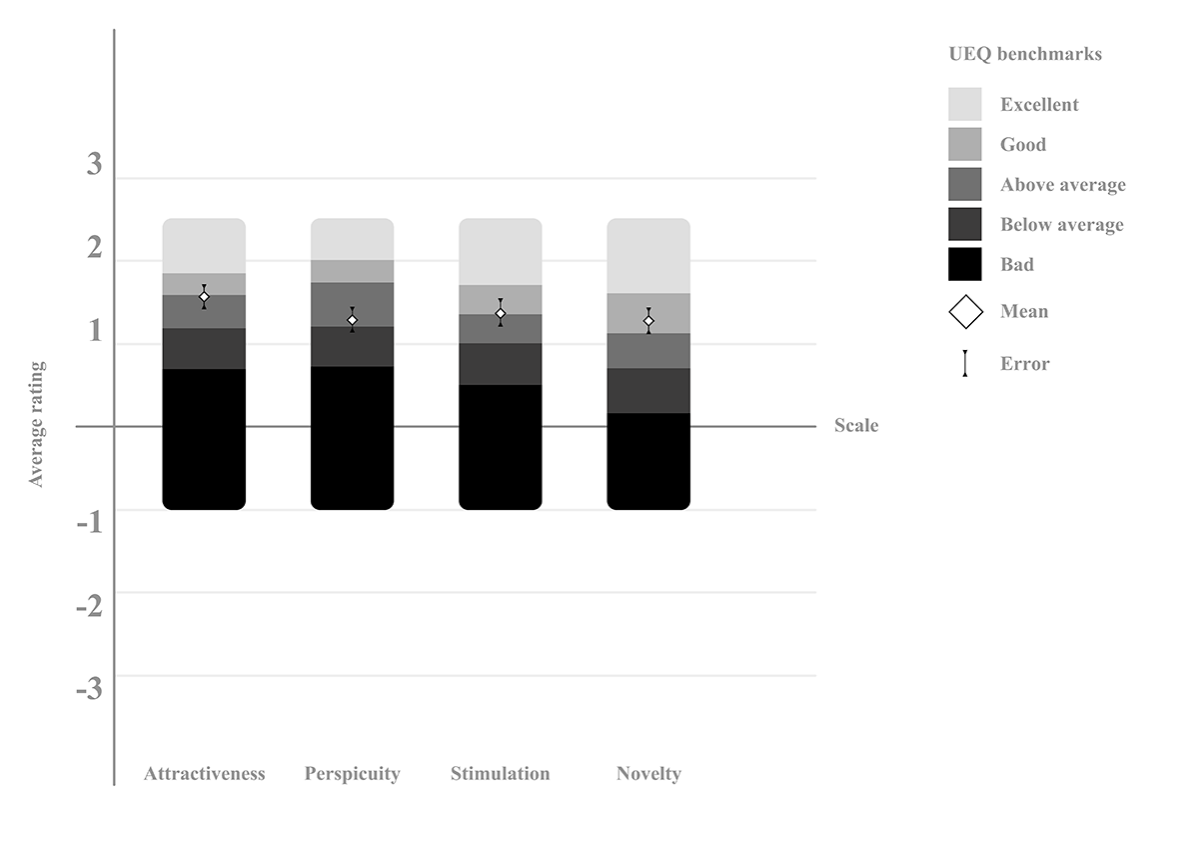
UX evaluation results of the futur.move mHealth service by study participants using the UEQ, compared to a dataset of 468 product reviews, analyzed with the UEQ Data Analysis Tool Version 12. Error bars represent the 95% CI. mHealth: mobile health; UEQ: User Experience Questionnaire; UX: user experience.

The UEQ benchmark refers to the mean scores and SD. According to the UEQ benchmark results shown in Figure 3, futur.move falls into the “good” category for hedonic quality (stimulation and novelty scales). The attractiveness and perspicuity scales are in the “above average” category. A score is considered “good” if it is among the top 25% in the benchmark dataset and “above average” if 25% of other products score better. Therefore, the overall quality of futur.move can be classified as good according to the UEQ. However, none of the scale scores fall into the “excellent” category, which includes the top 10% of results, indicating room for improvement.

#### Evaluation of Individual Features

**Figure 4:**
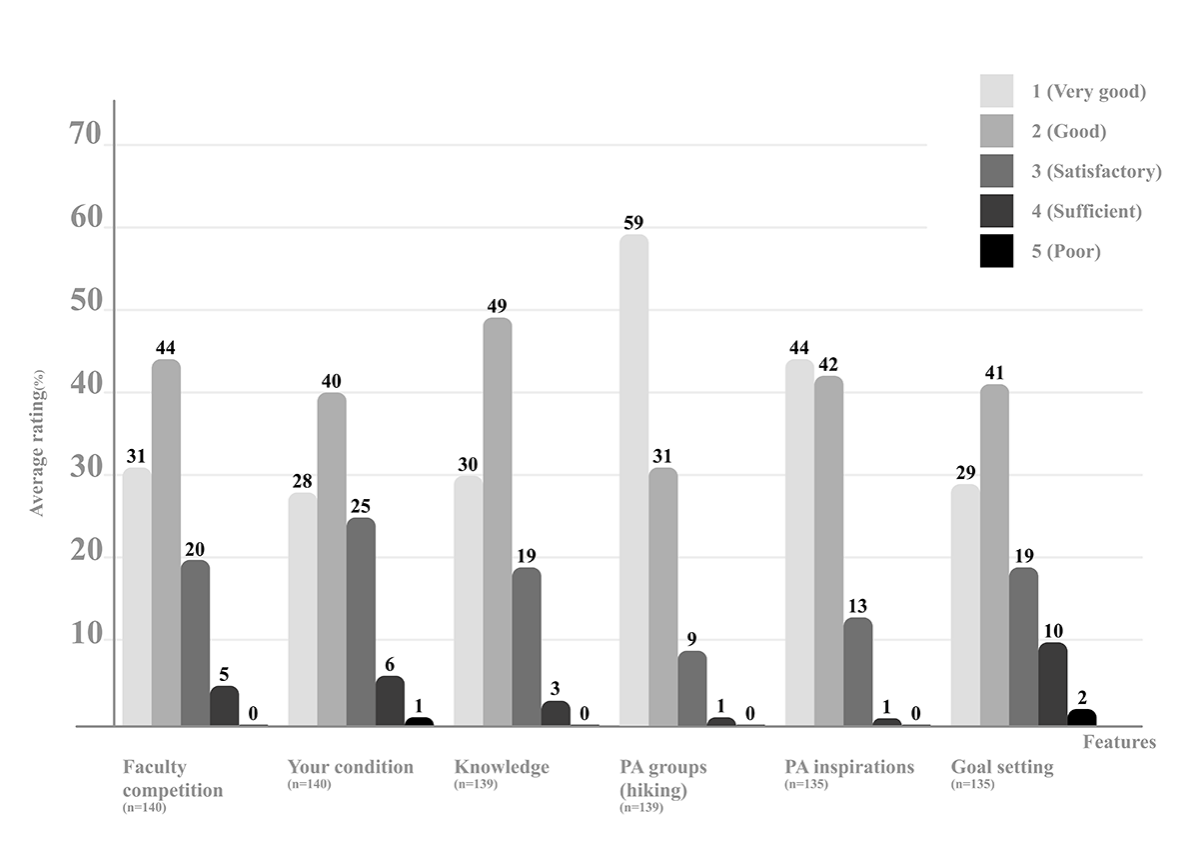
Average ratings of individual features of the futur.move mHealth service by study participants. Each feature was evaluated on a scale from 1 (very good) to 5 (poor). mHealth: mobile health; PA: physical activity.

When rating the individual features of futur.move, at least 68% (95/142) of participants rated all features with a score of 2 or better (Figure 4). The PA features (PA groups and PA inspirations) received the highest scores, with 90% (126/139) and 86% (116/135) of participants rating them with a score of 2 or better, respectively. Only a small number of respondents rated 2 features with a score of 5 (very poor): “your condition” (1/142, 0.7%) and “goal setting” (3/142, 2%).

#### Overall Grade

Overall, 105 (74%) of 142 participants gave futur.move an overall grade of 2 (n=97, 68%) or better (n=8, 6%). No grade lower than 4 was awarded (grade of 3: n=29, 20%; grade of 4: n=8, 6%). The average grade for the overall evaluation of futur.move was 2.3 (German grading scale, where 1 is the best grade and 6 the worst); a grade of 2 corresponds to a “good” rating.

#### Correlation Analysis

A summary table of the correlation analysis results can be found in Multimedia Appendix 4. To analyze the direction of correlations calculated with η, corresponding bar charts were used. These are also included in Multimedia Appendix 4.

The correlation analysis reveals a weak to moderate relationship between adherence to WHO guidelines for PA and the UX evaluation using the UEQ subscale perspicuity (η=0.232, *P*=.04). Adherence to WHO guidelines is associated with less favorable UX ratings.

Additionally, 2 weak but significant relationships were identified between age and the UEQ subscales perspicuity (Kendall τb=0.132, *P*=.03) and stimulation (Kendall τb=0.144, *P*=.02). Higher age was associated with more favorable evaluations of futur.move on both subscales.

A significant moderate relationship was also found between gender and the UEQ subscale novelty (η=0.363, *P*=.004), with women providing higher ratings for novelty compared to men.

No significant influence was observed for the variable prior experience with apps for PA on the UX evaluation using the UEQ.

### Qualitative Findings

#### Overview

**Figure 5:**
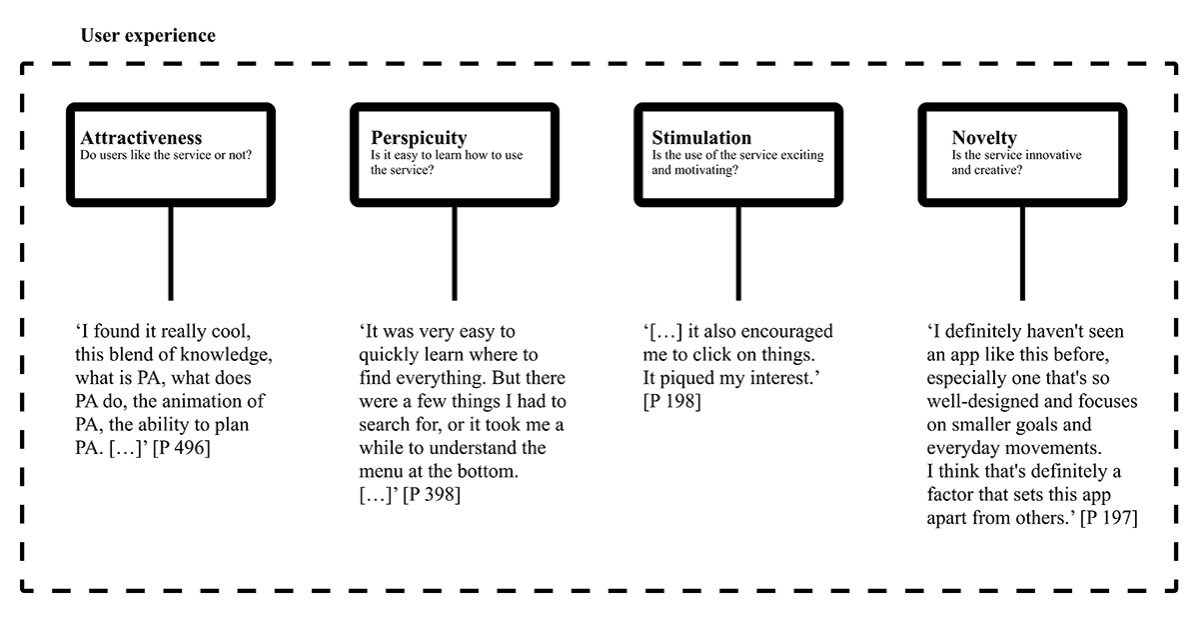
Overview of user experience categories for the futur.move mHealth service from the qualitative content analysis of 4 focus group discussions, structured according to UEQ subscales with illustrative anchor quotations. mHealth: mobile health; P: participant; UEQ: User Experience Questionnaire.

Four focus group discussions were conducted with a total of 23 participants. The main categories corresponded to the four scales of the UEQ: (1) attractiveness, (2) perspicuity, (3) stimulation, and (4) novelty. Figure 5 shows an overview of the UX dimensions with an exemplary anchor quote representing each category. The quotes mentioned in the text can be found in Multimedia Appendix 5.

#### Attractiveness

Most participants liked futur.move overall and had fun when using it (quote 1 in Multimedia Appendix 5). Some participants were particularly attracted by the variety of features (quotes 2 and 3 in Multimedia Appendix 5). However, few individuals found the platform to be less attractive. According to the participants, this was due to enduring a recent injury, living away from the university (quote 4 in Multimedia Appendix 5), or undertaking part-time studies (quote 5 in Multimedia Appendix 5). Many participants highlighted the networking opportunities with other students and the links to PA opportunities in the region (quotes 3 and 6 in Multimedia Appendix 5).

Most participants thought the “physical activity inspirations” were particularly attractive because of their diversity (quote 7 in Multimedia Appendix 5), perceived as a variety of offers for different needs. One participant thought it was particularly positive that the PA options were low-threshold; that is, they did not always entail sport but general PA such as a walk around the city (quote 8 in Multimedia Appendix 5). Most participants found the group activities attractive, even though other perspectives were also considered (quotes 9 and 10 in Multimedia Appendix 5). One person emphasized that the group recommendations were also a good way for first-year students to make contacts upon arrival in a new city (quote 10 in Multimedia Appendix 5). When using a digital service, the physical presence was also discussed. One participant particularly liked that futur.move offers the opportunity to meet in person (quote 11 in Multimedia Appendix 5). Most participants thought the “knowledge” feature was attractive. In particular, the variety of media was mentioned as appealing (quote 12 in Multimedia Appendix 5). However, details such as the structure of the texts also contributed to the perceived attractiveness (quote 13 in Multimedia Appendix 5). Many of the participants liked the “goal setting” feature in general. One of the reasons given was the possibility to set small, realistic goals (quote 14 in Multimedia Appendix 5). Several focus groups discussed appropriate target periods (quote 15 in Multimedia Appendix 5) and manual data entry (quote 16 in Multimedia Appendix 5).

Some of the participants thought it was attractive to compete with others in a faculty competition (quote 17 in Multimedia Appendix 5), while others would rather avoid such competition (quote 18 in Multimedia Appendix 5). One person cited a lack of connection to their own faculty as a reason (quote 19 in Multimedia Appendix 5).

In terms of the attractiveness of individual features, the option to design one’s own avatar elicited mixed feedback from participants. While 1 person thought it was appealing (quote 20 in Multimedia Appendix 5), another did not find the choice appealing at all (quote 21 in Multimedia Appendix 5).

Most participants were very positive about the design of futur.move (quote 22 in Multimedia Appendix 5). One participant particularly emphasized the design of the “knowledge” feature, which was perceived as clean (quote 23 in Multimedia Appendix 5). One person had a good feeling about the overall service but perceived a design break in the “your condition” feature (quote 24 in Multimedia Appendix 5). The color scheme of the service was rarely discussed, and apart from 1 comment on the color scheme in the “your condition” feature, was perceived as positive (quote 25 in Multimedia Appendix 5). The choice of images was particularly appealing to some respondents (quote 26 in Multimedia Appendix 5).

#### Perspicuity

Most participants described the platform as clear, tidy, and not too packed. The font, large headings, and organized layout contributed to this (quote 27 in Multimedia Appendix 5). Most features were also found to be easy to understand. Most participants reported that they found their way around quickly, although it took some time to get used to the system in the beginning (quote 28 in Multimedia Appendix 5). While certain aspects of the tasks required exploration, participants found the navigation easy to learn and were able to use it quickly (quote 29 in Multimedia Appendix 5).

In contrast, not all participants understood the diagrams in the “your condition” section or the points system for the “faculty competition” (quotes 30 and 31 in Multimedia Appendix 5). In 1 focus group, the comprehensibility of the navigation buttons in particular was discussed and perceived as somewhat difficult to comprehend (quote 32 in Multimedia Appendix 5).

#### Stimulation

Overall, the design and content of the service caught the interest of most participants and stimulated them to explore the content (quote 33 in Multimedia Appendix 5). In this context, individual feedback raised the question about the long-term motivation to use the service (quote 34 in Multimedia Appendix 5).

The “physical activity” feature received a lot of positive feedback, with the variety of activities seeming to be a key advantage. Not all activities appealed to everyone (quote 35 in Multimedia Appendix 5), but there was something for everyone (quote 7 in Multimedia Appendix 5). Most participants were particularly positive about the possibility of using futur.move to become physically active in the first place or even to initiate groups themselves (quote 36 in Multimedia Appendix 5). The “knowledge” feature also appealed to many participants, partly because of the variety of media and the design of the contributions (quote 37 in Multimedia Appendix 5). Many participants thought the “goal setting” feature was motivating, especially because small goals could be set and big goals could be broken down (quotes 38 and 39 in Multimedia Appendix 5). Opinions differed on the period length to achieve a target and whether the tool would be used during implementation (quotes 40 and 41 in Multimedia Appendix 5).

The “faculty competition” feature was discussed by participants. While some felt motivated by the competitive nature of the event (quote 42 in Multimedia Appendix 5), some participants did not find it appealing (quote 18 in Multimedia Appendix 5). Furthermore, the focus groups discussed the possibility of providing information about one’s own mood or having PA data displayed in the “your condition” feature: while some found this feature helpful, others thought it was uninteresting or even problematic to confront them with their own performance (quotes 43, 44, and 45 in Multimedia Appendix 5). Designing an avatar was a less motivating feature of the service to use for some participants (quote 46 in Multimedia Appendix 5).

#### Novelty

Participants indicated that they were particularly attracted by the creative design of the service (quote 47 in Multimedia Appendix 5) and the “fresh” headings (quote 48 in Multimedia Appendix 5). Some participants thought the service was new (quote 49 in Multimedia Appendix 5). It was not the content itself that was described as new, but its combination as an infrastructure service (quote 50 in Multimedia Appendix 5). The fact that the content was already familiar to some participants from other contexts was also a reason for some to perceive the service as less new (quote 51 in Multimedia Appendix 5).

## Discussion

### Principal Findings

The evaluation of the UX of futur.move using the UEQ revealed generally positive results, with attractiveness receiving the highest ratings. Compared to the UEQ benchmark, all scores were at least “above average.” However, all ratings still leave room for improvement. It has to be taken into account that despite rating options between –3 and +3, real-world applications typically produce lower values, with averages unlikely to exceed +2 or go below –2 [40]. “Your condition” and “goal setting” were less favorably rated, suggesting these areas are less engaging or useful for users. This feedback provides valuable insights for targeted enhancements to increase user satisfaction and engagement.

Qualitative results complemented the quantitative findings, reflecting the positive feedback on the service’s content and design. Participants particularly valued the personalization features, as well as the social and the variety of PA options, which enhanced their overall enjoyment. However, critical feedback identified areas for improvement, notably the manual data entry process and difficulties in comparing progress with other users.

The correlation analysis provides additional insights into potential areas for improving UX, though the findings should be interpreted with caution. A weak to moderate correlation was found between adherence to WHO guidelines for PA and the UEQ subscale perspicuity, contradicting that individuals who meet WHO guidelines for PA provide higher UX ratings for futur.move using the UEQ. As none of the other subscales showed any associations with adherence to the WHO guideline, this is probably a chance finding, as the relationship cannot be explained conceptually.

Interestingly, older students rated the UX higher on perspicuity and stimulation compared to younger students. These findings point to a need for better understanding what motivates the core target group of students aged 20-30 years to engage with the service and how to make it more intuitive for them. Although age appears to have a weak influence overall, these insights can guide refinements to improve engagement with this age group.

The observation that women perceive futur.move as more novel than men suggests that women rate UX higher, specifically in the subscale novelty. This finding emphasizes the importance of tailoring innovative features to better engage men and other genders, ensuring that the service effectively appeals to the diverse target audience. Addressing these issues could significantly enhance usability and overall UX. The analysis of the qualitative data by PA, gender, and age showed no additional insights.

### Comparison With Prior Work

Our study expands the existing literature by being to our knowledge the first to investigate the UX of an mHealth prototype in an early development phase using a combined qualitative and quantitative approach. It provides valuable insights into the strengths and weaknesses of the prototype, along with practical recommendations for further development.

Previous studies on UX in mHealth services aimed at promoting PA primarily focused on fully programmed apps in long-term studies, with a predominant emphasis on usability aspects [44-47]. For example, 1 study evaluated UX aspects using the UEQ without incorporating qualitative methods [48]. In contrast, our study integrates qualitative and quantitative UX methods in an early development phase to derive key insights into participatory development and its impact on UX.

The evaluation of **futur.move** revealed strengths in usability, personalization, and design, with the ease of use being particularly notable after a brief familiarization period. At the same time, weaknesses such as the need for manual data entry were identified, which could negatively affect long-term usage [16,29]. These findings align with studies emphasizing the importance of automated tracking features, intuitive navigation, and the critical role of personalization options [16,29,49].

The investigation of social comparison features revealed individual preferences. While some participants found the “faculty competition” motivating, others expressed a negative attitude toward competition, highlighting the diversity of user values. A systematic review recommends using competitive elements sparingly and in combination with other features [16]. The results of other studies suggest that intergroup competition approaches are beneficial [50,51].

Gender and age correlate with UX ratings. Women tended to evaluate the aspect of novelty more positively than men. One explanation could be that men are generally more frequent users of technology [52] and tend to score higher on measures of technology acceptance and competence [53,54]. This could explain why men perceive futur.move as less novel, as they may already be familiar with a wider range of similar offerings. Conversely, women often place greater emphasis on the design and user-friendliness of an application [55], which could positively influence their evaluation of its novelty. These findings highlight the importance of considering gender-specific preferences in UX design. For example, future development could focus on creating a balance between functionality and aesthetic appeal to meet the expectations of both genders. Additionally, efforts to better understand how men and women perceive and engage with novel features in mHealth apps could further optimize the UX of futur.move.

Research indicates that younger people are more likely to use smartphone apps for health-related purposes. For instance, as early as 2016, one-third of students surveyed in a study were already using mHealth apps [56]. Empirical evidence also shows that interest in technology tends to decrease with age among older adults [57]. Interestingly, in our study, older students rated the perspicuity and stimulation provided by futur.move more favorably. This could be attributed to differences in preferences, as older individuals may value ease of navigation and straightforward information over other features. While younger users generally engage more frequently with health apps, older adults may respond more positively to specific UX elements when tailored to their needs. Identifying these aspects should be a focus in future development.

### Limitations

In this study, a convenience sample of students was used. Participants were not selected based on their PA habits or need for PA in the university context. Due to initial reluctance from students to participate, they were engaged through personal networks within the student community. Not all courses of study and faculties of the university were covered. This recruitment process has led to a strong representation of health students in the sample. It can be assumed that some participants are already aware of health-related issues and the importance of PA for health. Therefore, the generalizability of the results to the entire student community may be limited.

In this study, 74% of the participants were female, similar to other mHealth intervention studies aimed at improving PA among students [49,58,59]. A study found that female students are also more likely to engage in health promotion activities on campus than males [14]. The participants’ age and BMI were similar to other studies [58-60]. Nevertheless, BMI has limitations as it does not account for body composition and can lead to health risk misestimation [61]. Self-reported height and weight can also cause BMI underestimation [62]. For this reason, BMI is not used in this study to refer to the students’ physical health. The service does not provide recommendations to students based on BMI. Future evaluation of the service should consider possible underestimations of self-reported data, especially concerning the effectiveness of the service, and include objective measurement methods.

Due to the presentation of the prototype in Figma, not all of the developed content of the service could be fully experienced. As a click dummy, a clickable prototype with limited functionality, the ability to make all activities of the service tangible is limited. For example, push notifications, which will be part of the service, could not be evaluated in this format. The evaluation of activities within the service was indirect and could not be directly tested in vivo. As futur.move was evaluated in a cross-sectional survey, the results provide insights into how students perceived its use but do not give information on the actual use of the service. A longitudinal evaluation of the programmed service is needed to collect data on user behavior and the effects of the service on PA behavior and motivation in long-term use. However, this evaluation requires not only a programmed service but also its implementation in everyday campus life, even if only on a trial basis.

### Implications

Future research should include analyses focused on the values and needs of younger students and men. These findings should then be incorporated into the further development of the service to better address and meet these groups’ specific requirements.

The service’s clarity, noted by most participants, confirms the successful layout of the application on which the usability of the service was based. Nonetheless, feedback on comprehensibility, such as navigation buttons, should be addressed for usability. The manual data entry process requires further review with the target group, as it was negatively received and could impede long-term use. Data tracking through devices might offer a solution here. Further investigation is also needed for competitive elements such as the faculty challenge.

Despite some criticism, the overall evaluation of futur.move is positive. futur.move meets a substantial proportion of the diverse needs of the target group, underscoring the importance of adaptability.

The next step is to assess whether using futur.move actually improves students’ PA. Thus, a longitudinal study should be carried out to assess real-world usability, long-term adherence, effectiveness in regards to PA, and other health-related outcomes and correlations with academic success.

Motivating students to participate in the development process remains challenging. Although 60% of surveyed students expressed interest in health programs [14], recruiting participants has been difficult. This suggests that a desire for the service does not necessarily lead to active participation in its design. Therefore, future studies should place greater emphasis on understanding the incentives and motivations that drive involvement.

Based on our findings, the UX evaluation methods used in this study could be adapted for other populations, such as individuals with chronic pain, obesity, or cardiovascular disease, to align with their needs and values in designing digital services or mobile apps that promote PA.

### Conclusions

The results of this study provide relevant information on how to further develop futur.move as a multidimensional, tailor-made, and participatory mHealth service to enhance PA among university students in hybrid spaces. By emphasizing personalization—a crucial factor also identified in prior research that showed the limitations of one-size-fits-all approaches [49]—futur.move aligns well with the diverse needs of its target audience.

Overall, the UX evaluation yielded positive results for the prototype. Students particularly appreciated its personalized content, engaging social features, and thoughtful design elements. However, the UX evaluation, complemented by qualitative feedback from focus group discussions, identified 2 specific features that need significant improvement to better meet user expectations. Additionally, reducing manual data entry emerged as a critical area for optimization, as its current implementation may hinder adherence and long-term user engagement.

While correlations between UX ratings and demographic variables were found to be weak to moderate, they warrant further investigation to better address the diverse characteristics of the student population. These findings may provide essential guidance for future development steps.

Building on these results, the next phase will refine the prototype by addressing the identified areas for improvement and implementing it as a smartphone app. This app will be tested and evaluated within the university context to ensure it effectively supports students in increasing PA while maintaining a high standard of UX.

## Appendix

Multimedia Appendix 1 CHERRIES for the futur.move mHealth service. mHealth: mobile health.

Multimedia Appendix 2 Screenshot of the customizable dashboard of the futur.move mHealth service as viewed on a smartphone in English. mHealth: mobile health.

Multimedia Appendix 3 Task sheet for user testing to evaluate the user experience of the futur.move mHealth service in German and English. mHealth: mobile health.

Multimedia Appendix 4 Table of correlation analysis results for user experience evaluation of futur.move mHealth service using UEQ subscales, including graphical interpretation. mHealth: mobile health; UEQ: User Experience Questionnaire.

Multimedia Appendix 5 Illustrative quotations from focus group discussions on the user experience of the futur.move mHealth service to support qualitative findings, presented in German and English. mHealth: mobile health.

## Abbreviations

CHERRIES: Checklist for Reporting Results of Internet e-Surveys
EHIS-PAQ: European Health Interview Survey–Physical Activity Questionnaire
HAWK: University of Applied Sciences and Arts Hildesheim/Holzminden/Göttingen
mHealth: mobile health
PA: physical activity
UEQ: User Experience Questionnaire
UX: user experience
WHO: World Health Organization

## Data Availability

The datasets generated and analyzed during this study are available from the corresponding author upon reasonable request.
