# Supplementary figures and images for: User Experience With a Personalized mHealth Service for Physical Activity Promotion in University Students: Mixed Methods Study
Source: JMIR Form Res. 2025 Mar 28;9:e64384. doi: 10.2196/64384 (PMC11992504; doi:10.2196/64384)

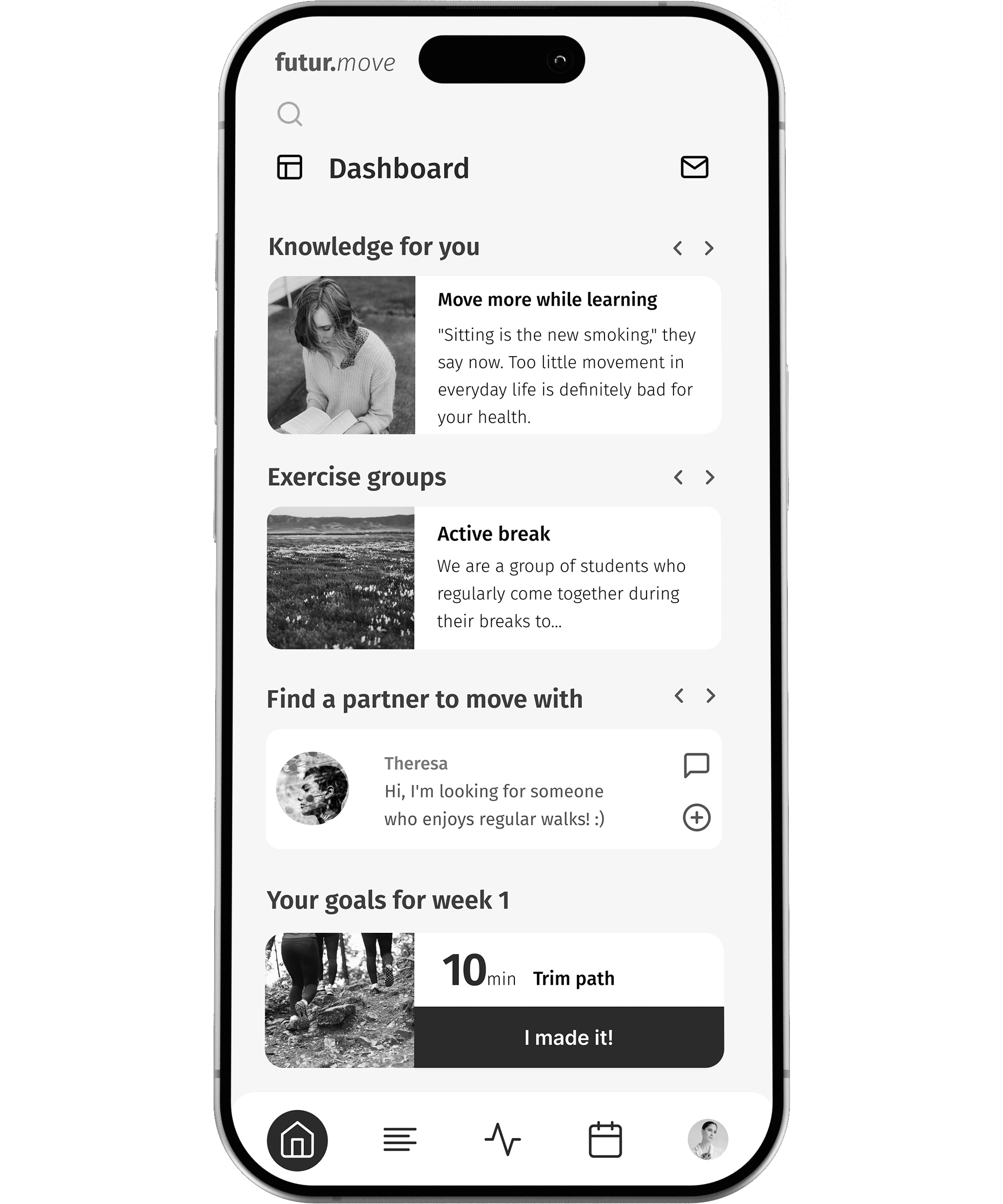

Supplement: Multimedia Appendix 2 [file formative_v9i1e64384_app2.png]
