# Supplementary material for: User Experience With a Personalized mHealth Service for Physical Activity Promotion in University Students: Mixed Methods Study
Source: JMIR Form Res. 2025 Mar 28;9:e64384. doi: 10.2196/64384 (PMC11992504; doi:10.2196/64384)
Supplement: Multimedia Appendix 3 [file formative_v9i1e64384_app3.pdf]

futur.*move*

Feedbackbogen

Deine ID:

Aufgabe 1

Du möchtest wissen, wie deine Fakultät im Bereich körperliche Aktivität im Vergleich zu anderen Fakultäten abschneidet. Gehe zum entsprechenden Bereich. Findest du den Vergleich und die anstehende Belohnung motivierend?

Ich habe den beschriebenen Bereich...

☐ gefunden. ☐ nicht gefunden.

Den Vergleich finde ich...

☐ motivierend. ☐ nicht motivierend.

Die Belohnung finde ich...

☐ motivierend. ☐ nicht motivierend.

Wie einfach viel dir das Erfüllen der Aufgabe?

Sehr einfach	☐	☐	☐	☐	☐	Sehr schwer
	**1**	**2**	**3**	**4**	**5**	

Wie gefällt dir der Bereich allgemein?

Sehr gut	☐	☐	☐	☐	☐	Sehr schlecht
	**1**	**2**	**3**	**4**	**5**	

Aufgabe 2

Du nutzt die Plattform schon eine Weile und möchtest sehen, wie sich deine Stimmung und deine Aktivität im Laufe der Zeit verändert haben. Gehe in den entsprechenden Bereich. Findest du die Angaben die du dort machen kannst sinnvoll und aussagekräftig?

Ich habe den beschriebenen Bereich...

☐ gefunden. ☐ nicht gefunden.

Die Inhalte finde ich...

☐ sinnvoll. ☐ nicht sinnvoll.

Die Inhalte finde ich...

☐ aussagekräftig. ☐ nicht aussagekräftig.

Wie einfach viel dir das Erfüllen der Aufgabe?

Sehr einfach	☐	☐	☐	☐	☐	Sehr schwer
	1	2	3	4	5	

Wie gefällt dir der Bereich allgemein?

Sehr gut	☐	☐	☐	☐	☐	Sehr schlecht
	1	2	3	4	5	

Aufgabe 3

Es wird ständig über körperliche Aktivität und Bewegung gesprochen. Schaue, wo und wie man sich auf der Plattform rund um das Thema Bewegung informieren kann.

Ich habe den beschriebenen Bereich...

☐ gefunden. ☐ nicht gefunden.

Die unterschiedlichen Formate (Podcast, Video, Artikel) finde ich...

☐ ansprechend. ☐ nicht ansprechend.

In diesem Bereich nach für mich relevanten Inhalten zu suchen kann ich mir...

☐ vorstellen. ☐ nicht vorstellen.

Aufgabe 3

Wie einfach fiel dir das Erfüllen der Aufgabe?

Sehr einfach ☐ 1 ☐ 2 ☐ 3 ☐ 4 ☐ 5 Sehr schwer

Wie gefällt dir der Bereich allgemein?

Sehr gut ☐ 1 ☐ 2 ☐ 3 ☐ 4 ☐ 5 Sehr schlecht

Aufgabe 4

Du möchtest die Aktivität „Wandern“ ausprobieren und versuchst über die Plattform herauszufinden, ob es die Möglichkeit einer gemeinsamen Wanderung gibt und wann diese stattfindet.

Die Menschen treffen sich zur Wanderung am...

☐ Dienstag. ☐ Freitag. ☐ Samstag. ☐ weiß ich nicht.

Die Möglichkeiten sich Bewegungsgruppen anzuschließen finde ich...

☐ gut. ☐ nicht gut.

Die Möglichkeit eigene Bewegungsgruppen anbieten zu können finde ich...

☐ gut. ☐ nicht gut.

Wie einfach viel dir das Erfüllen der Aufgabe?

Sehr einfach ☐ 1 ☐ 2 ☐ 3 ☐ 4 ☐ 5 Sehr schwer

Wie gefällt dir der Bereich allgemein?

Sehr gut ☐ 1 ☐ 2 ☐ 3 ☐ 4 ☐ 5 Sehr schlecht

Aufgabe 5

In einem weiteren Bereich werden viele kleine und große Anregungen für körperliche Aktivitäten an verschiedenen Orten im Alltag gegeben. Findest du diesen?

Ich habe den Bereich mit den Bewegungsideen...

☐ gefunden. ☐ nicht gefunden.

Die vorgeschlagenen Ideen finde ich...

☐ hilfreich. ☐ nicht hilfreich.

Wie einfach viel dir das Erfüllen der Aufgabe?

Sehr einfach	☐ 1	☐ 2	☐ 3	☐ 4	☐ 5	Sehr schwer

Wie gefällt dir der Bereich allgemein?

Sehr gut	☐ 1	☐ 2	☐ 3	☐ 4	☐ 5	Sehr schlecht

Aufgabe 6

Du möchtest planen, dich mehr zu bewegen. Wo auf der Plattform kannst du dich darum kümmern, dass du dich gezielt und regelmäßig mehr bewegst? Wenn du den Bereich gefunden hast, erstelle ein neues Vorhaben und besuche die Detailansicht von Woche 1.

Ich habe den beschriebenen Bereich...

☐ gefunden und konnte mir ein neues Ziel setzen. ☐ nicht gefunden.

☐ gefunden aber konnte mir kein neues Ziel setzen.

Ich habe die Detailansicht von Woche 1...

☐ gefunden. ☐ nicht gefunden.

Wie einfach viel dir das Erfüllen der Aufgabe?

Sehr einfach	☐ 1	☐ 2	☐ 3	☐ 4	☐ 5	Sehr schwer

Wie gefällt dir der Bereich allgemein?

Sehr gut	☐ 1	☐ 2	☐ 3	☐ 4	☐ 5	Sehr schlecht

futur.*move*

Feedback form

Your ID:

Task 1

You want to know how your faculty performs in the area of physical activity compared to other faculties. Go to the relevant feature. Do you find the competition and the upcoming reward motivating?

I have ______________ the described feature.

☐ found ☐ not found

The competition is...

☐ motivating ☐ not motivating.

The reward is...

☐ motivating. ☐ not motivating.

How easy was it fulfilling the task?

very easy ☐ 1 ☐ 2 ☐ 3 ☐ 4 ☐ 5 very difficult

How do you like the feature in general?

very much ☐ 1 ☐ 2 ☐ 3 ☐ 4 ☐ 5 not at all

Task 2

You have been using the platform for a while and would like to see how your mood and activity level have changed over time. Go to the relevant feature. Do you find the information you can enter there useful and meaningful?

I have ______________ the described feature.

☐ found ☐ not found

The content is...

☐ useful. ☐ not useful.

The content is...

☐ meaningful. ☐ not meaningful.

How easy was it to fulfil the task?

very easy	☐	☐	☐	☐	☐	very difficult
	**1**	**2**	**3**	**4**	**5**	

How do you like the feature in general?

very much	☐	☐	☐	☐	☐	not at all
	**1**	**2**	**3**	**4**	**5**	

Task 3

People are constantly talking about physical activity and exercise. See where and how you can find information about physical activity on the plattform.

I have ______________ the described feature.

☐ found ☐ not found

The different formats (podcast, video, article) are...

☐ appealing. ☐ not appealing.

I can ___________ searching for content in this feature.

☐ imagine ☐ not imagine

Task 3

How easy was it to fulfil the task?

very easy ☐ 1 ☐ 2 ☐ 3 ☐ 4 ☐ 5 very difficult

How do you like the feature in general?

very much ☐ 1 ☐ 2 ☐ 3 ☐ 4 ☐ 5 not at all

Task 4

You would like to try out the activity "hiking" and use the plattform to find out whether there is the possibility of a joint hike and when it will take place.

People meet up for a hike on...

☐ Tuesday. ☐ Friday. ☐ Saturday. ☐ I don't know.

I _________ the option to join physical activity groups.

☐ like ☐ dislike

I _________ the option to offer a physical activity group by myself.

☐ like ☐ dislike

How easy was it to fulfil the task?

very easy ☐ 1 ☐ 2 ☐ 3 ☐ 4 ☐ 5 very difficult

How do you like the feature in general?

very much ☐ 1 ☐ 2 ☐ 3 ☐ 4 ☐ 5 not at all

Task 5

Another feature offers many small and big suggestions for physical activty in various settings in everyday life. Can you find it?

I have ______________ the feature 'PA Inspirations'.

☐ found ☐ not found

The proposed inspirations are...

☐ helpful. ☐ not helpful.

How easy was it to fulfil the task?

very easy	☐ 1	☐ 2	☐ 3	☐ 4	☐ 5	very difficult

How do you like the feature in general?

very much	☐ 1	☐ 2	☐ 3	☐ 4	☐ 5	not at all

Task 6

You want to plan to be more physically active. Where on the platform can you make sure you move more regularly? Once you have found the area, create a new plan and visit the detailed view of week 1.

I have...

☐ found the described area and was able to set a new goal. ☐ not found the described area.

☐ found the described area but was not able to set a new goal.

I have ______________ the detailed view of week 1.

☐ found ☐ not found

How easy was it to fulfil the task?

very easy	☐ 1	☐ 2	☐ 3	☐ 4	☐ 5	very difficult

How do you like the feature in general?

very much	☐ 1	☐ 2	☐ 3	☐ 4	☐ 5	not at all
