# Supplementary material for: User Experience With a Personalized mHealth Service for Physical Activity Promotion in University Students: Mixed Methods Study
Source: JMIR Form Res. 2025 Mar 28;9:e64384. doi: 10.2196/64384 (PMC11992504; doi:10.2196/64384)

| Examined Relationship | Correlation Coefficient | Value | *P* value significance  (Pearson’s Chi-Square) |
| --- | --- | --- | --- |
|  |  |  |  |
| **WHO Compliance & UEQ Subscales** | |  |  |
| WHO Compliance & UEQ Attractiveness | Eta | .188 | .652 |
| WHO Compliance & UEQ Perspicuity | Eta | .232 | .042^a^ |
| WHO Compliance & UEQ Stimulation | Eta | .184 | .204 |
| WHO Compliance & UEQ Novelty | Eta | .077 | .502 |
|  |  |  |  |
| **Age & UEQ Subscales** | |  |  |
| Age & UEQ Attractiveness | Kendall-Tau-b | .114 | .063 |
| Age & UEQ Perspicuity | Kendall-Tau-b | .132 | .033^a^ |
| Age & UEQ Stimulation | Kendall-Tau-b | .144 | .020^a^ |
| Age & UEQ Novelty | Kendall-Tau-b | .067 | .280 |
|  |  |  |  |
| **Gender & UEQ Subscales** | |  |  |
| Gender & UEQ Attractiveness | Eta | .278 | .971 |
| Gender & UEQ Perspicuity | Eta | .120 | .689 |
| Gender & UEQ Stimulation | Eta | .345 | .346 |
| Gender & UEQ Novelty | Eta | .363 | .004^a^ |
|  |  |  |  |
| **App Experience & UEQ Subscales** | |  |  |
| App Experience & UEQ Attractiveness | Eta | .001 | .559 |
| App Experience & UEQ Perspicuity | Eta | .043 | .156 |
| App Experience & UEQ Stimulation | Eta | .046 | .990 |
| App Experience & UEQ Novelty | Eta | .020 | .301 |

Significant values alpha≤.05;

*^a^P*<.05

# Graphical Representation for Interpretation

## WHO Compliance & UEQ Perspicuity


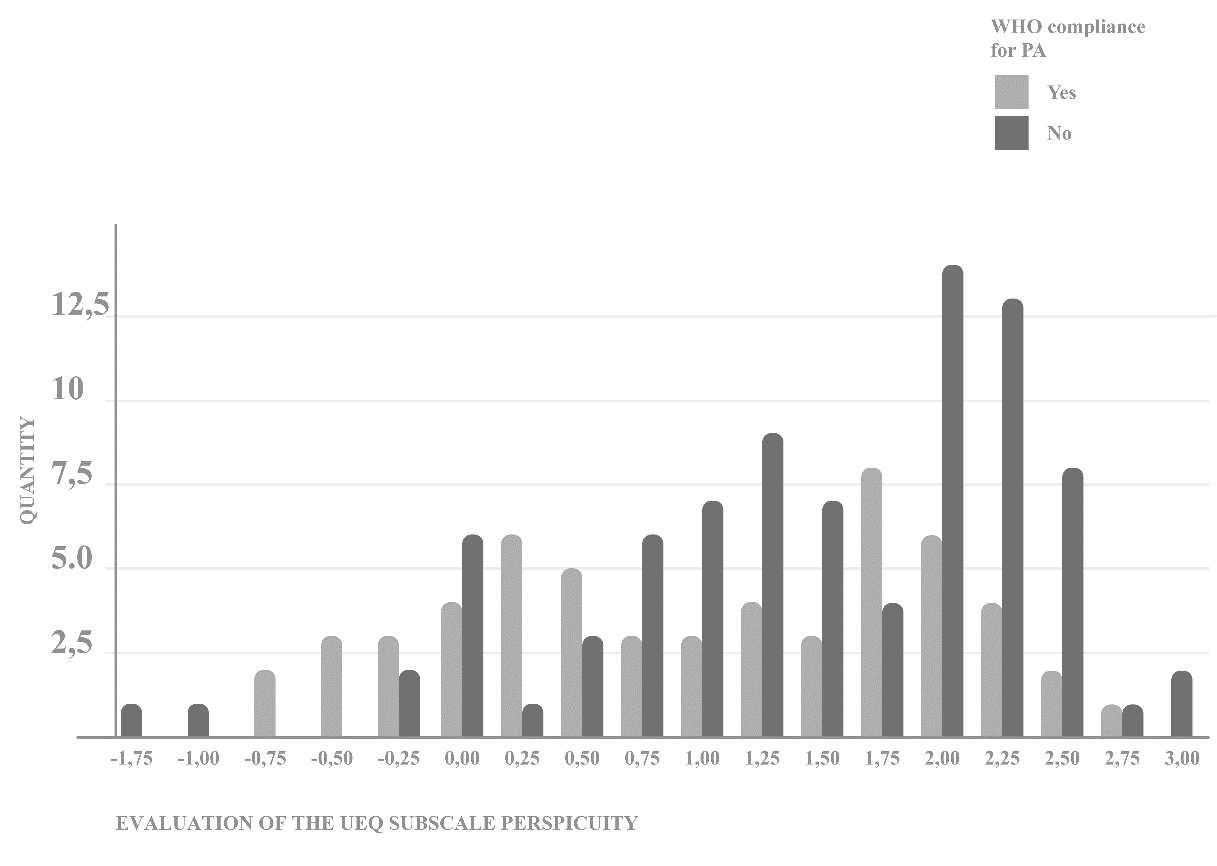


## Gender & UEQ Novelty


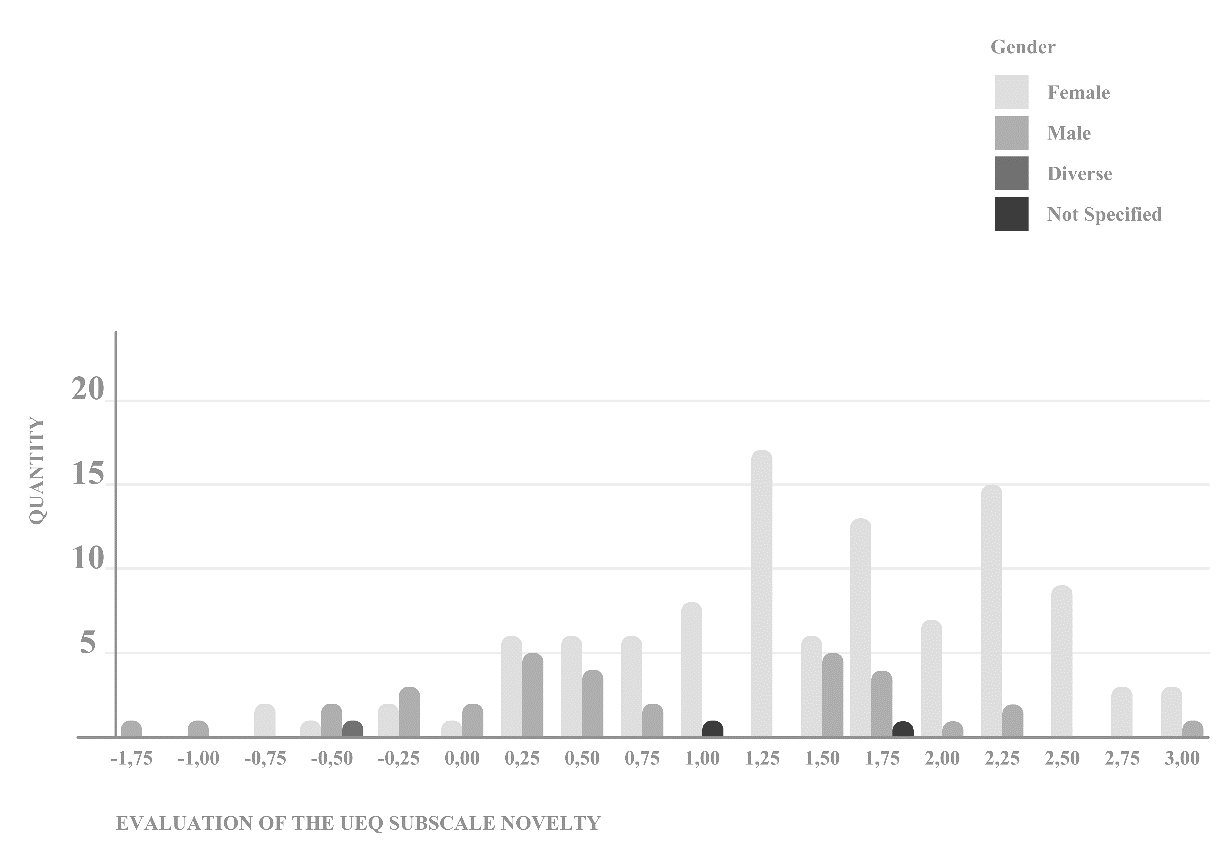

Supplement: Multimedia Appendix 4 [file formative_v9i1e64384_app4.docx]
