# Supplementary material for: User Experience With a Personalized mHealth Service for Physical Activity Promotion in University Students: Mixed Methods Study
Source: JMIR Form Res. 2025 Mar 28;9:e64384. doi: 10.2196/64384 (PMC11992504; doi:10.2196/64384)
Supplement: Multimedia Appendix 5 [file formative_v9i1e64384_app5.docx]

**Table S1:** Illustrative quotations from focus group discussions on the user experience of the futur.move mHealth service to support qualitative findings, presented in German and English.^a,b^

| Category | Illustrative quote |
| --- | --- |
| Attractiveness | 1. “[…] Mir hat es Spaß gemacht und ich fand es gut, dass man von Anfang an auswählen konnte, will ich mehr Achtsamkeit, will ich mehr Bewegung, will ich mehr Kraft.“ (TN 599)  *‘[…] I liked it and I liked that you could choose from the beginning: do I want more mindfulness, do I want more physical activity, do I want more strength?’ (P 599)*  2. “Ich fand es sehr cool, diese Mischung aus Wissen, (.) was ist Bewegung, was macht Bewegung, die Animation zur Bewegung, die Planbarkeit von Bewegung.“ (TN 496)  *‘I found it really cool, this blend of knowledge, what is PA, what does PA do, the animation of PA, the ability to plan PA. […]’ (PA 496)*  3. “[…] Das mit den Gruppen sich zu organisieren und zu treffen, fand ich mega cool. Und auch, dass es so eine große Vielfältigkeit gab. Also sei es jetzt Geocaching/ oder es war ja sogar Feiern gehen angegeben. Also dass sich das auch wirklich in die Freizeit integrieren lässt. Das fand ich total ansprechend. Auch so die Idee, eine neue Gruppe zu gestalten. Mir ist sofort eingefallen, mit Hund treffen und spazieren gehen als Beispiel.“ (TN 599)  *‘I thought it was really cool to organise and meet the groups. And also that there was so much variety. Whether it was geocaching or even going to parties. In other words, you could really integrate it into your free time. I really liked that. I also liked the idea of starting a new group. I immediately thought of getting together with a dog and going for a walk, for example.’ (P 599)*  4. “Ja, ich habe gerade eine Knieverletzung und bin in (Name der Stadt), studiere aber noch in (Name der Stadt). So gesehen ist die Plattform für mich halt jetzt nicht so der Renner, sag ich mal. […]” (TN196)  *‘Yes, I have a knee injury at the moment and I'm in (name of the city), but I'm still studying in (name of the city). From that point of view, the platform isn't really a big hit for me, I'd say.’ (P 196)*  5. „Also jetzt ich als im Masterstudium Studierende ist es halt, ich bin irgendwie für die Blockwochenenden in (Name der Stadt) und sonst halt nicht. (.) Da würde ich so etwas vielleicht jetzt nicht unbedingt nutzen oder diese App-Funktion nicht nutzen, weil es mir wahrscheinlich gar nicht so viel bringt […]“ (TN 493)  *‚Now, as a Master's student, it's just that I'm sort of in (name of city) for the block weekends and not otherwise. (...) I wouldn't necessarily use something like that or this app function because it probably wouldn't help me that much [...].’ (P 493)*  6. „Ich fand cool, dass auch externe Sachen da irgendwie einen Platz finden. Jetzt so mit/ da waren ja auch schon ein paar Vereine aus (Name der Stadt) aufgelistet.“ (TN 499)  *‘I thought it was cool that there was a place for outside stuff. Now with/ there were already some sports clubs from (name of city) listed.’ (P 499)*  7. „Und ich finde es ganz cool, dass es eben so verschiedene Sachen gibt und so voll verschiedene Angebote, sodass, glaube ich, jeder so ein bisschen was findet. (.) […]“ (TN 495)  *‘And I think it's really cool that there are so many different things and so many different offers, so I think everyone can find something. (.) [...]’ (P 495)*  8. „Da fand ich es mega cool, dass man auch, also wirklich nicht nur jetzt so Sportangebote, sondern vielleicht auch mal so Sachen wie, oh jetzt ist gerade, weiß nicht, Zirkus in der Stadt oder ist es Automeile oder irgendwas, was einen dann halt in die Stadt zieht, was aber ja irgendwie auch Bewegung ist, wenn man in die Stadt bummelt.“ (TN 599)  *‘I thought it was really cool that you could not only do sports but also things like, oh I don't know, there's a circus in town or a car show or something that attracts you to the city but is also a kind of physical activity as you walk around the city.’ (P 599)*  9. „Also ich finde es super interessant und sehr schön eigentlich, diese Gruppen zu haben, weil ich bin einfach insgesamt ein kommunikativer Mensch und mag das dann auch eher mit anderen was zu machen. […] Wobei ich auch Leute kenne, die Sport als was Privates sehen, Sport als für sich alleine. Die wollen alleine joggen, die machen ihr Yoga allein zu Hause und da interessiert die gar keine Gruppe. (....)“ (TN 598)  *‘So I think it's super interesting and actually really nice to have these groups because I'm just generally a communicative person and I like to do things with other people. [...] But I also know people who see sport as something private, sport for themselves. They want to go jogging alone, they do their yoga at home alone and they're not interested in a group at all. (....)‘ (P 598)*  10. „[…] Ich glaube, das ist eben für viele, die neu in eine Stadt kommen und mit so einem Angebot in Kontakt kommen, sicherlich auch eine gute Möglichkeit zum Socializen. Also dass man einfach vielleicht auch Gruppen findet, Freunde findet, in einer Stadt ankommt etc. (...) Um sich integrieren zu können, da wo man jetzt so neu lebt, […]“ (TN 496)  *‘[...] I think that for many people who are new to a city and come into contact with such an offer, it's certainly a good opportunity to make contacts. So that you can maybe find groups, make friends, arrive in a city, etc. (...) To be able to integrate where you're living so new [...].’ (P 496)*  11. „Ich fand auch gerade diese Möglichkeit, dass man sich zu Aktivitäten im realen Leben verabreden kann, darüber total schön.“ (TN 493)  *‘I also really liked the fact that you can arrange to meet up for real-life activities.’ (P 493)*  12. „Und auch cool fand ich, dass es verschiedene Möglichkeiten gab, sich über Sachen zu informieren, […]. Das fand ich total ansprechend, dass es da verschiedene Formate gab, also Videos und Podcasts zum Beispiel.“ (TN 599)  *‘And I also thought it was cool that there were different ways to find out about things [...]. I really liked that there were different formats, like videos and podcasts.’ (P 599)*  13. „Ja, also ich fand die Artikel, ich habe sie mir so überflogen, eigentlich sehr interessant. (.) Vor allen Dingen an diesem Wissen hat mich gecatcht, dass die halt gut aufgebaut waren. (.) […] Weil es war gut strukturiert, man hat Grafiken gehabt, die den Text aufgebrochen haben und auch fett gedruckte Worte, die einem so im Text geholfen haben, dass man einfach auch mal überfliegen kann. Und so wichtige Informationen herausfiltern kann, ohne wirklich den ganzen Text, so Fließtext lesen zu müssen. […]“ (TN 598)  *‘Yes, I found the articles, I skimmed through them, actually very interesting. (...) What I liked most about this knowledge was that it was well organised. (...) [...] Because it was well structured, there were graphics that broke up the text and words in bold print that helped you skim through the text. So you can pick out the important information without having to read the whole text. [...]‘ (P 598)*  14. „Ich glaube, das ist auch motivierend, weil die einzelnen Etappensachen, man sich die schön kleinstapeln kann, wenn man darauf Wert legt. […]“ (TN 399)  *‘I think it's also motivating because you can stack the stages if you want. [...]’ (P 399)*  15. „Also ich muss sagen, ich finde das eigentlich ganz gut, dass man so einen längeren Zeitraum hat, weil ich das Gefühl habe manchmal, wenn ich mir so Ziele setze, ich setze mir auch manchmal gerne zu hohe Ziele. Und wenn man einen längeren Zeitraum hat, dann hat man auch nicht direkt verloren so mäßig, weil man hat ja noch Zeit, das so aufzuholen in die Richtung. (.)“ (TN 198)  *‘I have to say, I think it's actually quite good that you have a longer period of time, because I sometimes have the feeling that when I set myself goals, I sometimes set myself goals that are too high. And if you have a longer period of time, then you don't immediately lose out, because you still have time to catch up in that direction. (.)‘ (P 198)*  16. „Was mich stören würde, ist, dass ich immer da reingehen muss und meine Aktivität selber aktiv da eintragen muss“ (TN 599)  *‘What would bother me is that I always have to go in there and actively enter my activity.’ (P 599)*  17. „Und auch die erste Aufgabe direkt mit der Vergleichbarkeit von Fakultäten, dass man da auch so einen kleinen interaktiven Anreiz hat. Das fand ich auch direkt einfach sehr, sehr gut. Innovativ tatsächlich und auch ein bisschen fesselnd. […]“ (TN 496)  *‘And also the first task directly with the comparability of the faculties, so that you also have a little interactive incentive. I thought that was really, really good. It was really innovative and also a bit engaging. [...]‘ (P 496)*  18. „Und was ich ganz spannend finde, ich habe mir das aber fast schon gedacht, dass einige total begeistert sind vom Thema Wettbewerb. Und für mich war das so, dass ich dachte, oh, nein, nicht noch ein Wettbewerb. Wir leben in einer so wettbewerbsorientierten Zeit. […].“ (TN 498)  *‘And what I find really exciting, but I almost imagined it, is that some people are really excited about the competition. And I thought, oh no, not another competition. We live in such a competitive time. [...].‘ (P 498)*  19. „Ich glaube, es ist gar nicht so krass für mich die Belohnung, die das was ausmachen würde, sondern eher, wenn es zum Beispiel anders aufgesplittet wäre, weil es ist so weit auseinander, die ganzen Fakultäten, mit denen hat man wirklich gar nichts zu tun, das heißt, so ein richtiger Konkurrenzkampf entsteht da nicht. […] also wenn das zum Beispiel in Fakultät Gestaltung so ein Ding zwischen den Kompetenzfeldern ist oder so, oder, so (..) dann wäre es vielleicht sogar spannend, weil man dann Bezug dazu hat. Ich glaube, der Bezug ist das, was so ein bisschen fehlt. (....)“ (TN 399)  *‘I don't think it's so much the reward that would make the difference for me, but rather if it was divided differently, for example because it's so far apart, all the faculties, you don't really have anything to do with them, which means there's no real competition. [...] So if, for example, in the Faculty of Design there was something like that between the departments or something like that (...) then it could even be exciting because then you have a connection to it. I think the connection is what's missing a bit. (....)‘ (P 399)*  20. „Aber ich muss auch sagen, tatsächlich mich catcht auch sowas schon mit, ich kann mein Avatar irgendwie anders gestalten. […]“ (TN 198)  *‘But I also have to say, I'm actually really caught up in it, I can sort of make my avatar different. [...]‘ (P 198)*  21. „Also ich finde mich da einfach gar nicht drin wieder, in den vorgeschlagenen, das sind ja vorgeschlagene Avatare. […]“ (TN 298)  *‘So I just don't see myself in the suggested avatars at all. [...]’ (P 298)*  22. „Mir hat das sehr gut gefallen, wie das gestaltet war.“ (TN 198)  *‘I really liked the way it was designed.’ (P 198)*  23. „Also ich fand diese Wissen-Section ganz cool und/ ja, ich weiß nicht, so gestalterisch passt das für mich halt komplett. (..) Also ich finde da muss halt nicht unbedingt so viel Schnickschnack oder so was, so krass viel Gestaltung oder so was drin sein. Ich finde das eigentlich ganz nett, so wie das so clean aufgebaut ist. […]“ (TN 595)  *‘So I thought this knowledge section was pretty cool and/or yeah, I don't know, in terms of design, that's totally my thing. (...) Well, I don't think there necessarily has to be so many bells and whistles or anything like that, so much design or anything like that. I actually think it's quite nice, the way it's so clean. [...]‘ (P 595)*  24. „Also mich spricht die Plattform, wie gesagt, an. Sie gibt mir irgendwie ein gutes Gefühl. […] Also jetzt von der Oberfläche und von der Optik her. Und ich habe aber manchmal das Gefühl, dass das Design so ein bisschen bricht. Also zum Beispiel, wenn ich auf meinen persönlichen Übersicht da gehe und dann gehe ich auf mein Aussehen und dann passt für mich, das, was da unten an Icons ist, nicht zu der Restoptik oder Gestaltung. […]“ (TN 298)  *‘Like I said, I like the platform. It gives me a good feeling somehow. [...] In terms of the interface and the look. But sometimes I feel like the design is a bit broken. For example, when I go to my personal dashboard, and then I go to my look, and then for me the icons at the bottom don't match the rest of the look or the design. [...]‘ (P 298)*  25. „Vielleicht an der einen oder anderen Stelle ein bisschen rosa. Also so bei diesen Stimmungen fand ich das noch okay. Aber irgendwann mal so ein Bereich, wo immer so drei Sachen, drei Wörter, ich weiß nicht mehr was es war und die waren dann auch in diesen, ich weiß nicht/ das ist persönliches Empfinden. (..)“ (TN 298)  *‘Maybe a little pink here and there. So with these moods I thought it was okay. But at some point there was an area where there were always three things, three words, I can't remember what they were, and they were also in these, I don't know/ that's a personal feeling. (..)‘ (P 298)*  26. „Was dann aber schön war, war, dass so viele Bilder da waren. Die haben das halt so freundlich gemacht und irgendwie so, dass man sich wohl gefühlt hat. (..) Genau. (...)“ (TN 495)  *‘But what was nice was that there were so many pictures. They made it so friendly and made you feel comfortable. (...) Exactly. (...)‘ (P 495)* |
| Perspicuity | 27. “Man hat sich nicht overwhelmed gefühlt. (..) Es war nicht zu viel so auf einen kleinen Platz gecramt. Es war alles sehr ordentlich, aber trotzdem nicht halt voll. Also man hat sich nicht überfordert gefühlt. Die großen Überschriften, Wissen, Bewegungsgruppen, Partnerinnen finden, (..) Gesamtübersicht der Ziele, all diese Sachen, die so gut gegliedert waren, fand ich ganz schön vom Layout her. Man konnte sich einfach gut durchfinden. […]” (TN 598)  *‚You didn't feel overwhelmed. (...) There wasn't too much crammed into a small space. It was all very tidy, but still not too crowded. So you didn't feel overwhelmed. The big headings, knowledge, exercise groups, finding partners, (...) general overview of the objectives, all these things that were so well organised, I thought the layout was really nice. It was easy to find your way around. [...]‘ (P 598)*  28. „Aber ansonsten habe ich jetzt ziemlich alles relativ schnell gefunden. Klar muss man sich am Anfang erstmal durchklicken, um zu sehen, wo überhaupt was ist. Aber ich würde sagen, ich habe auf jeden Fall alles nach einer Zeit sehr gut gefunden und auch wieder gefunden. (.)“ (TN 497)  *‘Other than that, I found everything relatively quickly. Of course, you have to click your way through to see where everything is. But I would say that after a while I definitely found everything very easily and then found it again. (.)‘ (P 497)*  29. „Genau, sonst fand ich die App ja auch sehr intuitiv. (.) Man war super schnell irgendwie in dem System drin, wie die App irgendwie funktioniert und wo man klicken muss, wenn man was Bestimmtes haben möchte.“ (TN 493)  *‘Exactly, otherwise I found the app very intuitive. (...) You quickly got to grips with the system, how the app worked and where to click when you wanted something.’ (P 493)*  30. „[…] mich hat es erstmal ein bisschen verwirrt, weil, also dieser obere Teil, wo man die Sachen anklicken kann, mit Schlaf, Sozial, Bewegung, hat sich irgendwie nicht unten widergespiegelt. Also ich habe dann nicht kapiert, warum soll ich das da oben anklicken, weil unten wird in den Schaubildern ja was anderes angezeigt, wenn man da unten weiterklickt auf Schlaf und Bewegung, was da noch kommt. […]“ (TN 299)  *‘[...] I was a bit confused at first because this top part where you can click on the things with sleep, social, exercise, was somehow not reflected at the bottom. So I didn't understand why I should click on that at the top because at the bottom of the graphs there's something else, if you click on sleep and exercise at the bottom, what else is there? [...]‘ (P 299)*  31. „Ja, ich hatte ja beim Profil gesehen von Kim, dass ja auch Punkte vergeben worden sind. Da hatte ich jetzt aber keinen Bezug gefunden, für was die stehen oder wonach die bewertet werden. (..)“ (TN 199)  *‘Yes, I had seen in Kim's profile that points were also awarded. But I couldn't find any indication of what they stood for or what they were rated on. (..)‘ (P 199)*  32. „Und was auch noch ein bisschen unklar war am Anfang, diese unteren vier Buttons, […]“ (TN 299)  *‘And something that was also a little unclear at first, these bottom four buttons, [...].’ (P 299)* |
| Stimulation | 33. “Ich fand das so recht clean und es hat auch dazu angeregt, drauf zu klicken auf die Sachen. Es hat mein Interesse geweckt.” (TN 198)  *‘I thought it was really clean and it also encouraged me to click on things. It got me interested.’ (P 198)*  34. “Was ich mich gefragt habe/ Also, ich habe es so gemerkt, umso länger ich mich damit/ umso länger ich mich durchklicke, umso mehr habe ich Lust, das mal auszuprobieren. Und ich habe mich voll gefragt, aber wie lang/ also wie die langfristige Motivation aussieht. Also, ob das erstmal so diese erste Begeisterung ist und dann nach so vier Wochen vergisst man, dass man diese App hat? […]” (TN 398)  *‘What I was wondering/ Well, I realised that the longer I clicked through it/ the longer I clicked through it, the more I wanted to try it. And I was really wondering how long/ what the long-term motivation would be. So whether it's this initial excitement and then after four weeks you forget you have this app? [...]‘ (P 398)*  35. „Also ich fand die Bewegungsideen an sich cool, bis auf diese eine Rubrik mit Zuhause oder so. (.) Da habe ich mir die Sachen durchgeklickt. Da war jetzt halt, wo ich so dachte, ich würde jetzt nicht irgendwie ein Parcours bei mir aufbauen. […] Aber die anderen fand ich an sich schon interessant auf jeden Fall. Dass man halt mal gucken kann und dann vielleicht was, vielleicht auch schon kannte und sich denkt, oh, das könnte ich aber mal wieder machen. […]“ (TN 499)  *‘Well, I thought the movement ideas themselves were cool, except for this one section with home or something. (...) I clicked through the things there. There was one that I didn't think I would do in my home. (...) But the others I found interesting. The fact that you can have a look and then maybe you know something, maybe you already knew something and you think, oh, I could do that again. [...]‘ (P 499)*  36. „Ja, auch, also was ich sehr schön finde daran an diesen Bewegungsgruppen, (.) dass man sich eben auch mit anderen Studierenden eigentlich connecten kann, mit denen man vielleicht so über jetzt die Hochschule gar keinen Kontakt hat, sondern das einfach nochmal, ja einfach über ein anderes Setting, nämlich über das Setting Bewegung oder sich gemeinsam bewegen, eine Möglichkeit entsteht, neue Leute auch kennenzulernen. (...) Und ja, auch die Möglichkeit, was Eigenes anzubieten, wenn man einfach selbst auch sagt, hey, ich habe da irgendwie ein Steckenpferd und das möchte ich gerne irgendwie teilen. (.) Und darüber eben auch Leute zu finden, die ein ähnliches Interesse eben wie ich selber habe, finde ich eine total schöne Idee. (.) […]“ (TN 493)  *‚Yes, what I also find very nice about these exercise groups (...) is that you can actually get in touch with other students with whom you might not have any contact at all through the university, but that simply, yes, simply through a different setting, namely through the setting of exercise or moving together, there is an opportunity to get to know new people. (...) And yes, also the opportunity to offer something of your own, if you simply say to yourself, hey, I've got a hobby and I'd like to share it somehow. (...) And I think it's a really nice idea to find people who have a similar interest to you. (.) [...]‘ (P 493)*  37. „Ich dachte auch noch, cool, dass es so multimedial ist. […] dass da was mit Text war, dass es Podcasts gab, dass irgendwie Verlinkungen zu Videos war. Dass man so ein bisschen Abwechslung hatte, das fand ich auch total cool, […]” (TN 269)  *‘I also thought it was cool that it was so multimedia. [...] that there was something with text, that there were podcasts, that there were sort of links to videos. I also thought it was really cool that there was a bit of variety [...].’ (P 296)*  38. „Ich finde, das gefällt mir auf jeden Fall am allerbesten von allem, weil ich das/ also, weil man da sehr viel Zeit/ oben steht, dann direkt, welche die aktuellen Ziele sind und so. Das finde ich cool. (..) Ich glaube, das ist auch motivierend, weil die einzelnen Etappensachen, man sich die schön kleinstapeln kann, wenn man darauf Wert legt. […]“ (TN 399)  *‘I think that's definitely my favourite thing of all, because I like that / well, because you have a lot of time / at the top, then you can directly see what your current goals are and so on. I think that's cool. (...) I think it's also motivating because you can stack the stages if you want to. [...]‘ (P 399)*  39. „[…] Ich fand es auch einen mega guten Punkt dieses, zu sagen, ich will sofort 40 Minuten mich mehr bewegen oder ich will das auf 12 Wochen oder sogar 24 Wochen aufgestapelt haben. (.)“ (TN 398)  *‘[...] I also thought it was a mega good point to say I want to do 40 minutes more exercise immediately or I want to stack it up over 12 weeks or even 24 weeks. (.)‘ (P 398)*  40. „[…] Also ich bin da auch eher so ein bisschen spontaner, dass ich mir jetzt auch keine krassen Ziele setze. Vielleicht so grobe Ziele, aber jetzt nicht irgendwie, dass ich an dem Tag die und die Minuten rausgehe oder so. […].“ (TN 595)  *‘[...] Well, I'm a bit more spontaneous in that I don't set hard and fast goals. Maybe rough goals, but not the kind where I go out for this number of minutes this day or something like that. [...].‘ (P 595)*  41. „Ich glaube, ich wüsste nicht, ob ich diese App eher für persönliche Ziele nutzen würde, sondern eher für dieses einfach machen. (..) […]“ (TN 598)  *‘I don't think I would use this app for personal goals, just to do it. (..) [...]’ (P 598)*  42. „Fand ich auch noch diesen Fakultätsvergleich/ Also ich meine, vielleicht liegt das darin, dass ich diesen Wettbewerbsansporn sowieso immer geil finde, mich mit anderen zu vergleichen im Sport. (.) Aber so zu sehen, so ja, meine Fakultät Gesundheit und Soziales steht an zweiter Stelle. Ich mache nochmal safe ein paar Schritte mehr, um die da so ein bisschen nach oben zu bringen. Und auch, dass man eine Party gewinnen konnte, super cooler Ansporn, also geile Motivation auch in der App, fand ich irgendwie.[…]“ (TN 599)  *‘I also found this faculty competition/ Well, I think maybe it's because I always find it cool to compare myself with others in sports anyway. (.) But if you look at it that way, yes, my Faculty of Health and Social Sciences is in second place. I'm sure I'll take a few more steps to push it up a bit. And the fact that you could win a party was also a super cool incentive, I thought it was a great motivation in the app too [...].’ (P 599)*  43. „Also ich glaube, so eine Übersicht, gerade über die Aktivität, dass man sieht, wie man sich steigert, ist schon sehr wertvoll. (.) Und auch, dass die Stimmung angezeigt wird. (..)“ (TN 299)  *‘So I think having an overview like that, especially of your activity, so you can see how you are improving, is very valuable. (.) And also that the mood is shown. (..)’ (P 299)*  44. „Aber, genau, ich habe auch den Teil, glaube ich, mit diesen Stimmungspunkten auch ein bisschen schlechter bewertet als die anderen, weil ich auch dachte, (.) och, würde ich das wirklich machen? (.) Und genau, ich habe zum Beispiel auch eine Uhr, die das auch tracken kann und dann glaube ich, wäre das niederschwelliger, das über die Uhr zu machen. […]“ (TN 296)  *‘But, exactly, I think I also rated the part with these mood points a little bit lower than the others, because I also thought (.) oh, would I really do that? (...) And exactly, I also have a watch, for example, that can track this, and then I think it would be easier to do it via the watch. [...]‘ (P 296)*  45. „[…] Also, wenn ich jetzt aktuell irgendeine Anzeige hätte, die mir über die Zeit anzeigt, wie gestresst meine Stimmung ist, das fände ich sehr ernüchternd. Ich weiß auch nicht, ob das mich daran ändern würde. Also weil es die Gesamtumstände sind. Ich glaube, das kann für Leute problematisch sein oder auch ein Trigger. […].“ (TN 399)  *‘So if I had some kind of display that showed me how stressed I was over time, I would find that very sobering. I don't know if it would change me. So because it's the overall circumstances. I think that can be a problem or a trigger for people. [...].‘ (P 399)*  46. “Und ich für meinen Teil bin jetzt nicht so, dass ich meinen Avatar groß aufpimpen muss. (.) Also ich finde, das an sich ist eine coole Idee. Ich glaube, das spricht vielleicht auch viele an, aber mich jetzt persönlich nicht so.“ (TN 196)  *‘And I'm not one to pimp my avatar too much. (.) So I think it's a cool idea in itself. I think it might appeal to a lot of people, but not to me personally.’ (P 196)* |
| Novelty | 47. „Ja, also ich habe mich für die Kreativität ausgesprochen. […] Und mir ist auch noch so die Illustrationen sind mir aufgefallen. Ich fand den Stil cool. Ja. (....)“ (TN 196)  *‘Yes, I was for creativity. [...] And I also noticed the illustrations. I thought the style was cool. Yeah. (....)‘ (P 196)*  48. „Also mir ging es auch so, dass ich es voll ansprechend fand, irgendwie auch wieder mit diesem Titel, das habe ich vorher schon gesagt. Also irgendwie finde ich, das ist so frisch irgendwie. […]“ (TN 494)  *‘I also found it really appealing, somehow even with this title, as I said before. Somehow I think it's so fresh. [...]’ (P 494)*  49. “Aber ja, kreativ fand ich es auf jeden Fall. Und ich fand es auch neu. Ich hab tatsächlich noch nie so eine App benutzt, außer mal so einen Mood-Tracker.“ (TN 198)  *‘But yeah, I definitely found it creative. And I also found it new. I've never really used an app like this before, apart from a mood tracker.’ (P 198)*  50. „Und ja, eigentlich, dass viele Sachen, die es schon gibt in anderen Apps, jetzt hier irgendwie so zusammenfließen, […]“ (TN 493)  *‘And yes, actually, a lot of things that already exist in other applications are kind of coming together here, [...].’ (P 493)*  51. „[…] Ich finde, mit den Zielen fand ich einmal ein bisschen, also an sich finde ich es cool, (..) aber ich habe das irgendwie schon in so vielen anderen Apps gehabt, dass ich mir da irgendwie so Ziele stecke, denen ich dann irgendwie hinterher hechel. […]“ (TN 299)  *‘[...] I think I found the goals a bit, well, I think it's cool in itself, (...) but I've had that in so many other apps, that I sort of set goals and then sort of chase them. [...]‘ (P 299)* |

^a^ The original German quotations have been translated into English. The English translation is in italics.

^b^ The information in brackets is the identification number of the focus group participant.
